# Reporting participation rates in studies of non-pharmacological interventions for patients with chronic obstructive pulmonary disease: a systematic review

**DOI:** 10.1186/2046-4053-1-66

**Published:** 2012-12-29

**Authors:** Ratna Sohanpal, Richard Hooper, Rachel Hames, Stefan Priebe, Stephanie Taylor

**Affiliations:** 1 Centre for Primary Care and Public Health, Blizard Institute, Barts and The London School of Medicine and Dentistry, Queen Mary University of London, 58 Turner Street, London, E1 2AB, UK; 2 Unit for Social and Community Psychiatry, Barts and The London School of Medicine and Dentistry, Queen Mary University of London, Newham Centre for Mental Health, London, E13 8SP, UK

**Keywords:** Patient participation, Patient dropouts, Randomised controlled trial, Controlled clinical trial, Chronic obstructive pulmonary disease, Self-care, Self-management

## Abstract

**Background:**

Pulmonary rehabilitation (PR) and self-management (SM) support programmes are effective in the management of patients with chronic obstructive pulmonary disease (COPD), but these interventions are not widely implemented in routine care. One reason may be poor patient participation and retention. We conducted a systematic review to determine a true estimate of participation and dropout rates in research studies of these interventions.

**Methods:**

Studies were identified from eight electronic databases including MEDLINE, UK Clinical Trial Register, Cochrane library, and reference lists of identified studies. Controlled clinical trial studies of structured SM, PR and health education (HE) programmes for COPD were included. Data extraction included ‘participant flow’ data using the Consolidated Standards of Reporting Trials (CONSORT) statement and its extension to pragmatic trials. Patient ‘participation rates’ (study participation rate (SPR), study dropout rate (SDR) and intervention dropout rate (IDR)) were calculated using prior participation definitions consistent with CONSORT. Random effects logistic regression analysis was conducted to examine effects of four key study characteristics (group vs. individual treatment, year of publication, study quality and exercise vs. non-exercise) on participation rates.

**Results:**

Fifty-six quantitative studies (51 randomised controlled trials, three quasi-experimental and two before-after studies) evaluated PR (n = 31), SM (n = 21) and HE (n = 4). Reports of participant flow were generally incomplete; ‘numbers of potential participants identified’ were only available for 16%, and ‘numbers assessed for eligibility’ for only 39% of studies. Although ‘numbers eligible’ were better reported (77%), we were unable to calculate SPR for 23% of studies. Overall we found ‘participation rates’ for studies (n = 43) were higher than previous reports; only 19% of studies had less than 50% SPR and just over one-third (34%) had a SPR of 100%; SDR and IDR were less than or equal to 30% for around 93% of studies. There was no evidence of effects of study characteristics on participation rates.

**Conclusion:**

Unlike previous reports, we found high participation and low dropout rates in studies of PR or SM support for COPD. Previous studies adopted different participation definitions; some reported proportions without stating definitions clearly, obscuring whether proportions referred to the study or the intervention. Clear, uniform definitions of patient participation in studies are needed to better inform the wider implementation of effective interventions.

## Background

Chronic obstructive pulmonary disease (COPD) is a large and increasing public health problem. The disease is expected to be the third leading cause of death by 2020 [1], and is already the most costly respiratory disease in Europe, estimated at €38.7 billion annually [2]. In the UK, COPD affects 2.8 million people although only 0.8 million are diagnosed with the condition [3]. COPD is an irreversible, potentially disabling, lung disease characterised by fatigue and breathlessness and is associated with episodic ‘exacerbations’ [4,5] that lead to unscheduled health care [6]. An individual with COPD may experience significant functional and psychological limitations disrupting their normal routine and further preventing adherence to medical regimes, dietary changes, exercise and smoking cessation, which can further worsen the condition [3].

Pharmacological treatment only constitutes part of COPD care. Non-pharmacological interventions, such as pulmonary rehabilitation (PR) including patient education, exercise training, psychosocial support and nutritional intervention complement pharmacological therapy [7]. Self-management (SM) programmes have been promoted as another non-pharmacological intervention for helping people with chronic conditions [8]. Self-management refers to an ‘individual’s ability to manage symptoms, treatment, physical and psychosocial consequences and lifestyle changes inherent in living with a chronic condition. Efficacious self-management encompasses ability to monitor one’s condition and to effect the cognitive, behavioural and emotional responses necessary to maintain a satisfactory quality of life’ [9]. The aim of a PR programme is to reduce symptoms, improve functional performance, increase participation and reduce health care costs [10]. Using Bourbeau’s [11] definition, SM programmes are aimed at teaching the skills needed to perform a specific medical regimen and to achieve health behaviour modification. National health policy guidelines and charities strongly support and recommend the delivery of PR, and provision of SM education and support to help patients with COPD to better manage their condition [3,6] and reduce cost to health services. There is a huge need amongst patients for more education on the disease, management of breathlessness and exacerbations [3,12].

Despite this, a large number of people are unable to access these interventions [13,14]. Although there is evidence of considerable benefit from PR [14,15], only 1 to 2% of patients are able to access PR programmes because of patient factors, lack of referral from primary care practitioners and lack of infrastructure for provision of PR [14]. In a recent review Bjoernshave [15] questioned whether this benefit could be extrapolated to the entire PR target population as the patients in studies (including 26 articles) were not representative of the target populations. There is also limited implementation of SM programmes for COPD patients in practice. This may be because of the limited evidence of effectiveness of studies of SM programmes for COPD patients [13,16]. Effing’s review [16] noted that synthesising evidence of effectiveness of SM from studies was difficult due to heterogeneity in interventions, COPD populations, follow-up times and outcome measures. Another reason for lack of implementation could be the poor patient participation and retention frequently reported in the literature on such interventions [6]. Reduced patient participation or high attrition in studies of PR or SM programmes for COPD patients may affect the generalisability of the study findings to the target population.

There are varied reports of poor participation and high dropout rates amongst studies. Studies report that only about 34% of participants attend after being referred to PR [17] and uptake figures of between 33% and 39% have been reported from COPD outpatient clinics [18]. A recent review (including 11 articles) [19] reported that the proportion of referred participants who failed to attend PR at all ranged from 8.3% to 49.6%, and the proportion of PR dropouts ranged from 9.7% to 31.8%. Our own study of a COPD disease-specific SM programme [20] identified poor study participation (only 23%). Attrition may also be a problem but only one study out of fourteen in a review [16] of COPD SM education reported a dropout rate (30.4%).

Furthermore, we found large discrepancies between our calculation of study participation rates in some studies [21-23] and those reported by other authors [24,25]. Other studies report participation rates in interventions based on different stages of participant flow before recruitment, for example, participation rates calculated from numbers referred into the study [17] or from the numbers screened for the study [26]. In addition, it is unclear from some studies [18,24] which level of participant flow was used to calculate the participation or dropout rate and whether the proportion reported refers to the study or the intervention. This lack of uniformity or clarity all leads to further confusion about actual patient participation rates in studies and interventions.

To address the apparent problem of poor participation and retention, and to identify ways it could be improved, we undertook a systematic review to identify actual levels of participation and attrition reported in randomised and non-randomised studies evaluating non-pharmacological interventions providing self-management support to COPD patients. We hypothesised that the following study characteristics, recruitment process, patient characteristics, intervention characteristics and study quality may influence study participation rates and used random effects logistic regression analysis to explore this.

## Methods

### The search

A comprehensive search strategy was developed from other SM systematic reviews (COPD SM education, uptake of cardiac rehabilitation, SM in musculoskeletal pain) [8,16,27,28], MeSH headings and free text words were used. Relevant studies were identified from searching eight electronic biomedical science databases, and UK Clinical Trial Research registers (1984 to January 2011). We also searched the Cochrane library for systematic and meta-analysis reviews. The reference lists of all identified reviews, and published and unpublished grey reports by organisations that develop and deliver SM programmes for COPD patients in the UK were examined for relevant studies. Only English language papers were included. Additional file 1 presents details on the search strategy and the terms searched.

### Study selection criteria

We included all randomised controlled trials (RCTs), as well as non-randomised studies including before-after studies. Interventions included structured self-management (SM) programmes, pulmonary rehabilitation (PR) programmes, self-care (SC) programmes and health education (HE) programmes for adults with COPD. Interventions could be either group-based or targeted at individuals and conducted in any setting, for example, outpatients, inpatients, participant’s home, GP surgery, community, or remote (web-based or telephone) or a combination of these settings. Intervention delivery could be by a health professional, or a trained lay person or both. Conference abstracts, surveys and interventions that only included exercise and only SM plans or action plans were excluded.

We obtained full papers of studies identified as potentially eligible based on titles and abstracts. The full copies of the potentially eligible papers were obtained to assess whether the studies met the pre-specified inclusion criteria. If additional information was needed, we contacted the corresponding authors of the study.

### Definitions of participation

**Figure 1:**
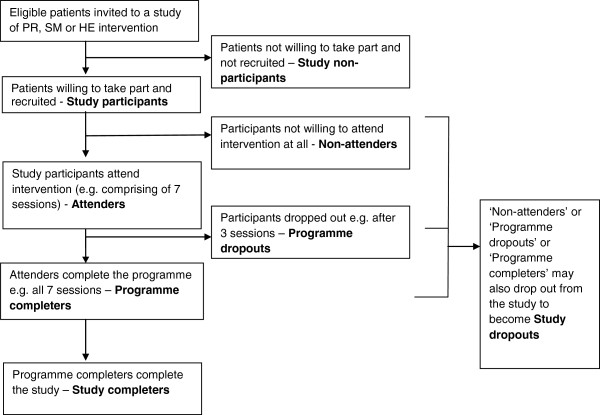
Illustration of patient participation definitions.

For the purpose of the review, we adopted the following definitions (Figure 1):

• ‘Study participation’- eligible patients taking part in a study of pulmonary rehabilitation (PR) or self-management (SM) or health education (HE) intervention and ‘study participants’ - patients that take part in the study.

• ‘Study non-participation’- not taking part in a study of PR or SM or HE intervention and ‘study non-participants’ - patients who do not take part in the study.

• The ‘study participant’ in the intervention arm of the study can be subdivided into an ‘attender’ - one who is exposed to at least part of the intervention (for example, attends at least one session) and a ‘non-attender’ - one who is not exposed to any part of the intervention (for example, does not attend any sessions of the intervention).

• The ‘attenders’ can be further divided into ‘intervention dropouts’ - those who drop out from the intervention and ‘intervention completers’ - those who complete the intervention.

• ‘Study completer’ - A ‘non-attender’, ‘intervention dropout’ or ‘intervention completer’ who completes the study.

• ‘Study dropout’ - A ‘non-attender’, ‘intervention dropout’ or ‘intervention completer’ who withdraws or is lost to follow-up from the study.

### Quality assessment

We included studies of any quality since we were interested in examining whether higher participation rates tended to be reported in higher quality studies. We appraised the quality of both randomised and non-randomised (including before-after) studies using the criteria generated by Downs and Black [29]. The Downs and Black checklist for quality assessment was selected as it has been developed to use with both randomised and non-randomised studies and is recommended as being suitable for use in systematic reviews [30,31]. Validity and reliability on the original version of the checklist was conducted by experienced epidemiologists and statisticians and a revised version produced [29,31]. Further assessment of the revised checklist showed that Quality Index had high internal consistency, good test-retest (r = 0.88) and inter-rater (r = 0.75) reliability and good face and criterion validity (0.90) [29].

The checklist allows an overall score for study quality to be reported as well as scores for each of the subscales. The question on power was simplified to a simple check whether the study had conducted a statistical power calculation. The maximum score achievable for each of the subscales was: 11 for reporting, 3 for external validity (an area which has been ignored in all checklists of RCTs), 7 for internal validity - bias in the measurement of the intervention and outcomes, and 6 for internal validity - confounding (selection bias), totalling to maximum score of 27.

### Data extraction

A data extraction form was developed and piloted for particular questions to be addressed by the review, and final versions were used to compile summary tables of the data and quality classification. Data extraction included study characteristics (study design, study setting, study eligibility criteria, recruitment process), population characteristics, intervention characteristics, definition of intervention completion, and study outcomes that included participation data.

The patient participation data (before and after recruitment) was extracted from studies by referring to the Consolidated Standards of Reporting Trials (CONSORT) participant flow diagram [32] and the checklist suggested by extension of the CONSORT statement for reporting of pragmatic trials [33]. We extracted the following data, ‘numbers of potential participants identified’ ‘numbers assessed for eligibility’, ‘numbers eligible’^a^, ‘numbers included (and randomised or not randomised)’ to all intervention groups, ‘numbers received allocated intervention’, ‘numbers did not receive allocated intervention’, ‘numbers lost to follow-up’, ‘numbers discontinued intervention’, and ‘numbers analysed for the primary outcomes’.

A second reviewer checked extraction and calculation of participation rate data from 10% of the studies sampled at random using a web-based random integer generator [34].

### Data analysis

**Figure 2:**
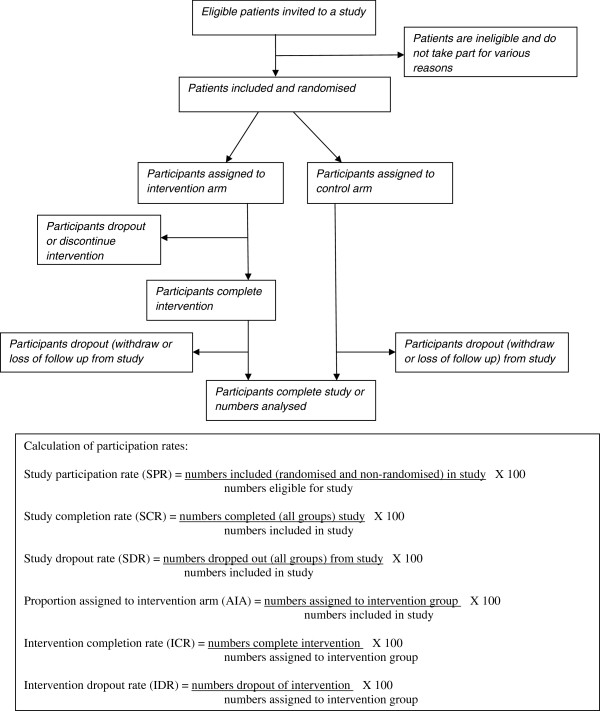
Calculation of participation rates.

Calculation of patient ‘participation rates’ from the extracted participation data was based on the adopted definitions (Figure 2). The study participation rate (SPR) was calculated based on ‘numbers included in study’ divided by ‘numbers eligible’. This method of calculation for SPR is consistent with wording used in extension of the CONSORT statement for reporting of pragmatic trials ‘… numbers were eligible for study of whom (%) agreed to participate’ [33]. Glasgow [35] also recommended expanding the criteria in the original CONSORT statement to include eight items on external validity, one of which was ‘report the participation rate among those eligible’.

For studies that reported both the number of eligible people and the number who were finally included, we determined participation rate with a 95% confidence interval calculated using a score method with a continuity correction [36]. We used random effects logistic regression, with participation of each individual as a binary outcome and a random effect of study, to estimate the effects of different study characteristics on participation rates. This amounts to a meta-regression of study results, and allows studies to be included even if their estimated participation rate is 100%.

Because of the relatively small number of studies with complete data, we chose to limit ourselves to looking at four study characteristics: year of publication (linear effect per year), quality score (linear effect per scale point), exercise vs. non-exercise intervention, and group vs. individual treatment (divided into three categories: individual, combined group and individual, and group). Only studies with complete data were included. There was heterogeneity between studies in COPD severity, but this variable was inconsistently reported and difficult to categorise, so was not selected for inclusion. Results are reported as adjusted odds ratio from a multivariable regression model including all four study characteristics.

Comparisons of what was reported by studies before and after publication of the CONSORT guidelines were made using chi-squared tests (or Fisher’s exact test where any expected frequency was <5).

## Results

**Figure 3:**
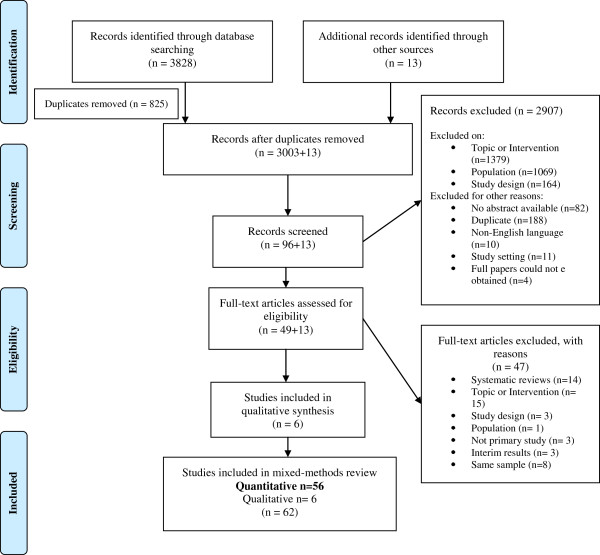
PRISMA flowchart showing process of search results for COPD studies.

We identified 3828 studies from the database search and 13 additional studies from other sources (Figure 3). After screening, 56 quantitative studies met our inclusion criteria, 51 RCTs, three quasi-experimental studies and two before-after studies. Thirty-one studies evaluated PR programmes, twenty-one evaluated SM programmes, and four studies evaluated HE programmes.

Study quality was variable. The overall quality of study reporting was good (9.9), external validity was low (1.3), internal validity was better amongst studies but, more bias was present in selection of study subjects (3.6) in comparison to bias in the measurement of the intervention and outcome (4.8). Less than half of the studies had conducted a power calculation. There was no clear pattern observed from the quality assessment that high quality studies had higher participation rates. The second reviewer checked a randomly selected 10% of data extractions and there was 100% agreement between the two reviewers.

### Reporting of participation data and calculation of participation rate

Additional file 2 provides information on patient participant flow (‘number of potential participants identified’, ‘numbers assessed for eligibility’, ‘numbers eligible’ for study, ‘numbers included (randomised or non-randomised)’ to all intervention groups, ‘numbers lost to follow-up’, and ‘numbers discontinued intervention’) and patient ‘participation rates’ - study participation rate (SPR), study dropout rate (SDR) and intervention dropout rate (IDR) by interventions of interest. Participant flow was poorly reported in all studies. Only nine (16%) studies reported ‘numbers of potential participants identified’ (4/31 PR, 4/21 SM and 1/4 HE) and twenty-two (39%) studies reported ‘numbers assessed for eligibility’ (12 PR and 10 SM). ‘Numbers eligible’ by studies was better reported, 43 (77%) studies, (21 PR, 19 SM and 3 HE). Fifty-six studies reported ‘numbers included (both randomised and non-randomised)’ in study, out of five non-randomised studies, two were before-after studies without a control group. Only seven (13%) studies recorded participant flow numbers right up to participant recruitment. Forty-one (73%) studies were published after the CONSORT statement in 2001. Better reporting of participant flow was seen in studies published in and after 2001 in comparison to studies published before 2001, ‘numbers of potential participants identified’ 9/41 (22%) vs. 0/15 (0%) (Fisher’s exact test *P* = 0.094); ‘numbers assessed for eligibility’ 18/41 (44%) vs. 4/15 (27%) (chi-squared = 1.37, df = 1, *P* = 0.24) and ‘numbers eligible’ 33/41 (80%) vs. 10/15 (67%) (chi-squared = 1.18, df = 1, *P* = 0.28).

Based on the data available, we were able to calculate SPRs for 43 studies. Half of the highest value in the range of proportions for SPR, SDR and IDR was taken as a cutoff value to show studies with participation rates above or below the chosen cutoff value. The SPR amongst studies of PR programmes ranged from 35 to 100% (that is, a cutoff value of 50%), only three (14%) studies having less than 50% SPR. In studies of SM programmes, SPR ranged from 23 to 100%, with four (21%) studies having less than 50% SPR. And amongst the three studies of HE programmes, SPR was 43%, 73% and 92%. Altogether for 43 studies (21 PR, 19 SM, and 3 HE) the SPR was less than 50% for only 8 (19%) studies with 12 (34%) studies reporting SPR of 100% (9 PR and 3 SM).

We calculated SDR for all 56 studies and IDR for all studies except for the two before-after studies (here the result of IDR and SDR was the same). Amongst PR studies, study dropout rates ranged from 0 to 59% (that is, cutoff value of 30%) with 27 (87%) studies having SDR of less than or equal to 30%. For studies of SM and HE programmes, the SDR ranged from 0 to 30% and from 11 to 21%. Overall, 52 (93%) studies had an SDR of less than or equal to 30%.

The IDR amongst studies of PR programmes ranged from 0 to 54%, 30 (97%) studies having IDR of less than or equal to 30%. Amongst studies of SM programmes, IDR ranged from 0 to 60% (that is, a cutoff value of 30%), 18/20 (90%) studies having less than 30% IDR. And in studies of HE programmes IDR ranged from 7 to 29%. Overall, IDR for 51/54 (94%) studies was less than or equal to 30%.

Although we were able to calculate the SDR and IDR, it was difficult to identify and differentiate between the number of participants who were lost to follow-up and participants who discontinued the intervention. We assumed that participants who dropped out of the study also dropped out of the intervention unless papers explicitly stated otherwise.

As SPR was calculated for 43 studies, 31/43 (72%) studies with SPR of >50%, had SDR of ≤30%. However, no obvious pattern could be deduced as 26/31 (84%) studies had not reported on participant flow data (‘potential participants identified’ and/or ‘numbers assessed for eligibility) before recruitment (Additional file 2).

**Figure 4:**
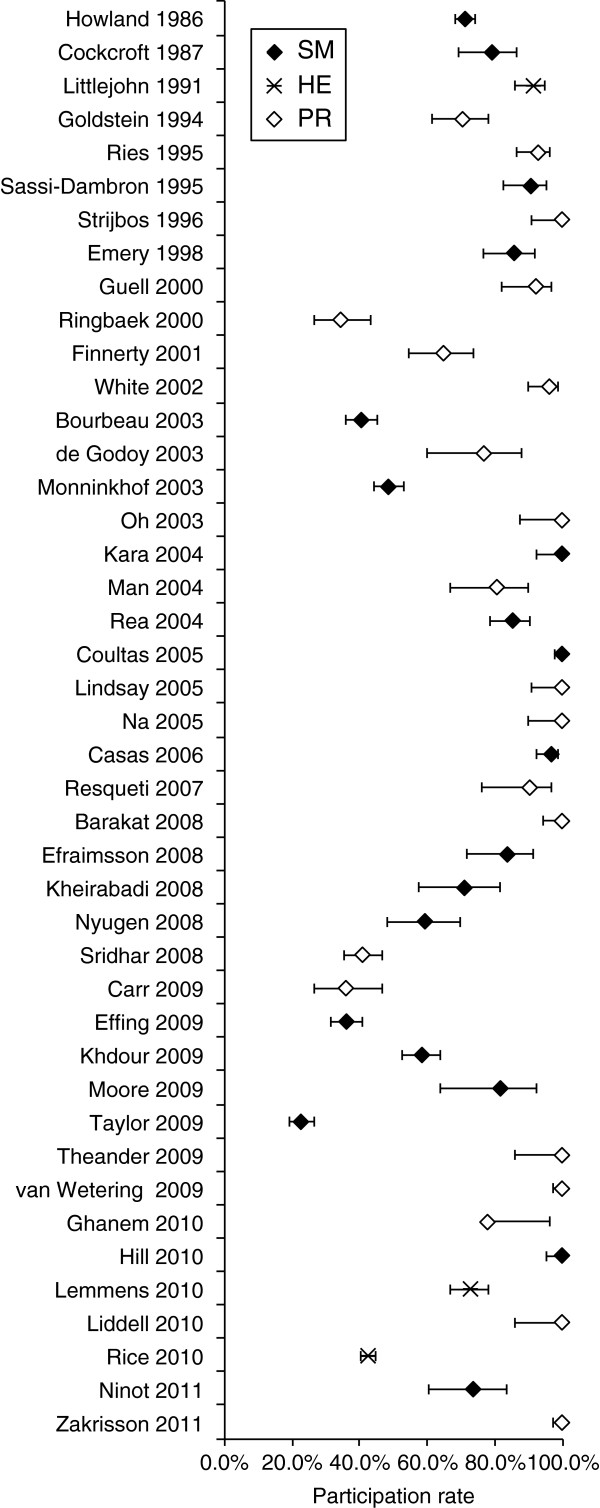
**Participation rates in different studies ordered by year of publication, and according to type of intervention: self management (SM), health education (HE), and pulmonary rehabilitation (PR).** Error bars show 95% confidence interval.

In the analysis of study characteristics, there was no evidence for effects of year of publication, study quality, exercise vs. non-exercise, and group vs. individual treatment on participation rate (Table 1). Figure 4 illustrates how year of publication had no effect on participation rate. Confidence intervals for effects were wide, and did not rule out the possibility of a five-fold increase in the odds of participation in exercise vs. non-exercise interventions, or a five-fold decrease in group vs. individual interventions.

**Table 1:** Odds ratios for participation according to study characteristics

**Variable**	**Odds ratio**	(**95%confidence interval**)	***P***	
Year	0.99	(0.92, 1.08)	0.891	
Quality score	0.85	(0.65, 1.10)	0.215	
Exercise intervention	1.55	(0.47, 5.07)	0.470	
Group intervention			0.506	(trend)
Individual	1.00	-		
Combination	0.17	(0.03, 0.81)		
Group	0.60	(0.17, 2.11)		

## Discussion

### Principal findings

Our review provides information on how randomised and non-randomised studies, including before-after studies, of interventions that help to improve SM in patients with COPD, report participant flow and the actual ‘participation rates’ amongst these studies. The reporting of participant flow amongst studies of the two main interventions (PR and SM) was generally incomplete but better reporting was seen in studies published in and after 2001 (the publication year of the CONSORT statement). Only 16% of studies reported ‘potential participants identified’, and slightly more than a third (39%) reported ‘numbers assessed for eligibility’. ‘Numbers eligible’ was better reported amongst studies (77%) but only seven (13%) studies reported on all levels of the participant flow before patient recruitment.

The SPR was not calculated for 13 (23%) studies due to lack of information on ‘numbers eligible’ for study. The SPR for the remaining 43 studies was higher than expected. Only eight (19%) studies had an SPR of less than 50%. Another unexpected finding was that 93% and 94% of the studies had an SDR and an IDR, respectively, of less than or equal to 30%. However, it was tricky to differentiate between ‘numbers lost to follow-up’ and ‘numbers discontinued intervention’. In addition, 31 (72%) of 43 studies with SPR of >50%, had SDR of ≤30% but no obvious pattern could be deduced because of the lack of reporting on participant flow data from 26 (84%) of the 31 studies.

### Comparison with other literature

Some studies of both pharmacological and non-pharmacological interventions and surgical interventions, have examined reporting of participant flow diagrams (CONSORT statement recommended) including type of information within the diagrams, in published studies identified from a single electronic database [37], in six high-quality [38] and four high-impact journals [39], with most journals endorsing the CONSORT reporting of participant flow [37]. These studies concluded that participant flow was poorly reported. In Toerien’s study [38] 40% of studies failed to report ‘numbers assessed for eligibility’. Meanwhile, only 39% of studies in our review reported ‘numbers assessed for eligibility’ but our study selection was not based on the quality of the journal. We found low reporting at this level of participant flow in studies perhaps because studies did not think it important to record numbers for external validity, they might not have considered that patients are part of the trial at that level or before randomisation and hence failed to record and report numbers at this level [38,40]. It has been acknowledged that studies of PR programmes do not include details or discuss adequately ‘numbers assessed for eligibility’ nor the refusal rate [15,25].

Regarding reporting ‘numbers of potential participants identified’ the aforementioned studies did not look at this level (this level is not included in the CONSORT flow diagram). A drawback of strictly designed RCTs may be limited generalisability as the focus is often to have homogenous groups of patients to limit individual variation [41]. A recent literature review [15] of PR programmes looked at reporting of sample selection in studies of PR programmes and only 12% of studies had reported the number of people contacted for the study. In our review, the proportion of studies that reported at this level was slightly higher (16%). Bjoernshave [15] explained the lack of recording at this level was because people with COPD are not normally recruited from prevalence studies as prevalence of COPD is difficult to estimate and recruitment normally takes place from clinics or outpatient settings. Nevertheless, if we attempt to record the total number of patients registered at a recruitment site ‘denominator’, this data can be utilised to help generalise the study findings to the target group [35].

Gross’s review [39] review found that only 43% of studies had reported ‘numbers eligible’. In our review, more studies had reported this (77%) perhaps because most of the studies were published in and after 2001. With numbers at this level and numbers recruited we were able to calculate the SPR, SDR and IDR and identify the actual patient ‘participation rates’ in studies of PR, SM and HE programmes for COPD patients. Only Keating’s [19] review has explored patient non-attendance and non-completion, but only in PR programmes and the reported proportions were from a mix of quantitative and qualitative studies. A cutoff value of 20% for SDR is regarded as acceptable according to a quality assessment checklist [42]. And less than a third (29%) of studies in our review had a SDR of >20%, which suggests that most studies in our review would have fulfilled this particular quality criterion. Similarly to other studies [37,38] we too experienced problems in clearly identifying or differentiating between reports of ‘numbers lost to follow-up’ and ‘numbers discontinued intervention’. A distinction needs to be made between these two types of attrition [40] to inform on implementation of interventions.

Based on our findings, previous reports of poor participation and retention in studies of PR and SM programmes [17-20] might not be justified. One explanation could be studies having different definitions for patient participation and thus the method of calculation of participation rates may have differed [17,26] or not having clear definitions making it difficult to identify if the proportions refer to the study or intervention [18]. Recent reviews [19,38] acknowledged that their studies gave varying definitions for ‘loss to follow-up’ and ‘non-completion’. Examples here show discrepancies in reports of participation rates: two studies [21,22] in our review (Additional file 2) had high SPRs of 71% and 93%, respectively but Young [25] reported, SPR of 34% and 36% for these two studies. On investigation it appears that Young calculated SPR from ‘numbers assessed for eligibility’ and not ‘numbers eligible’; Another study in our review [23] (Additional file 2) had a SDR of 29% and IDR of 18% respectively but Sabit [24] reported a dropout rate of 30% and it is unclear whether the proportion refers to the study or intervention.

### Limitations

We tried to identify numbers reported at each level of the participant flow from effectiveness studies. These studies may have decided to give more importance towards recording and reporting numbers for internal validity – a key feature of strictly designed or high-quality RCTs [41] rather than external validity. This finding is also acknowledged by the new Medical Research Council guidance on evaluation of complex interventions [43]. We need to be cautious of our findings of high participation rates as only a minority of studies reported on all levels of participant flow before recruitment and in some cases 100% of eligible patients were recruited without providing the whole recruitment picture.

### Implications for practice

Based on our findings of high study participation rates and low dropout rates in research studies of PR, SM and HE programmes, we would strongly endorse the active implementation of PR and SM programmes in routine care as patients with COPD are participating, attending and completing them. Despite notable evidence of benefit from studies of PR programmes [44] and some benefit from studies of SM programmes [16], in practice these programmes do not seem to be widely implemented or some actively running ones are closing down (Kennedy A. Personal Communication) thus reducing opportunities for patients who are suitable to attend and gain benefits from them. We also recommend that future research studies provide clear definitions when reporting patient participation, enabling a true estimate of patient ‘participation rates’ and avoiding confusion amongst readers.

To calculate ‘participation rates’, it is important for studies of PR, SM and HE programmes, to provide more information on patient participant flow. Incomplete reporting of patient recruitment data will affect external validity [15]. It is essential for studies to report these data to help health care professionals interpret the study results and to decide if the results could be applied to their patients [38,39].

Much focus on the implementation of non-pharmacological interventions has resulted in a shift, from conducting explanatory trials to pragmatic trials [41]. One of the features of pragmatic trials is that they tend to recruit a heterogeneous patient group all with the condition of interest to maximise the trial results to usual care settings [45]. To record and report a clear picture of the recruitment process, studies can utilise the checklist provided by extension of the CONSORT statement for reporting pragmatic trials [33]. Gross [39] stressed that studies should at least record and report ‘numbers eligible’ for recruitment. The addition of several boxes to the CONSORT flow diagram, before and after randomisation, has been recommended by Toerin [38], which may help to get a better assessment of generalisability, estimate a true non-participation rate, and to establish a true intervention effect.

## Conclusions

This systematic review has identified the actual levels of participation and dropout rates in research studies evaluating PR, SM and HE programmes for COPD patients. These studies should consider recording and reporting participant flow numbers more completely. Only 19% of studies had SPR of less than 50%. The SDR and IDR was less than or equal to 30% in the vast majority of studies. These findings negate previous reports of poor participation and retention in studies of PR and SM programmes. Possible explanations include studies using their own definitions for what constitutes patient participation in both the study and the intervention within the study, or studies, not stating definitions clearly, making it difficult to identify whether proportions reported refer to the study or intervention. Clear and uniform definitions will help to identify a valid estimate of patient participation rates in the study and the intervention and could promote the correct interpretation of studies and the implementation of effective interventions in routine care.

### Endnotes

^a^In five studies ‘numbers eligible’ was not clearly stated. So to calculate SPR in the five studies ‘numbers eligible’ were extracted the following way: reasons reported for not taking part in the study, between numbers assessed for eligibility and numbers included in study, were reported as numbers that declined to participate and numbers that were ineligible for the study. The numbers who declined to take part were added to numbers included in the study and were extracted as ‘numbers eligible’.

## Abbreviations

CONSORT: Consolidated Standards of Reporting Trials; COPD: Chronic obstructive pulmonary disease; HE: Health education; IDR: Intervention dropout rate; PR: Pulmonary rehabilitation; PRISMA: Preferred Reporting Items for Systematic Reviews and Meta-analyses; SDR: Study dropout rate; SM: Self-management; SPR: Study participation rate; RCTs: Randomised controlled trials.

## Competing interests

The authors declare that they have no competing interests.

## Authors’ contributions

RS participated in the conception and design, data extraction, analysis, interpretation of data and drafting the manuscript. RH was involved in revising the manuscript critically for important intellectual content. RH assessed the data extraction. SP and ST were involved in the conception and design and revising the manuscript critically for intellectual content. All authors read and approved the final manuscript.

## Supplementary Material

Additional file 1 Search strategy and search terms. Click here for file

Additional file 2 Participant flow data and participation rates of three interventions. Click here for file
