# Supplementary material for: Reporting participation rates in studies of non-pharmacological interventions for patients with chronic obstructive pulmonary disease: a systematic review
Source: Syst Rev. 2012 Dec 29;1:66. doi: 10.1186/2046-4053-1-66 (PMC3563605; doi:10.1186/2046-4053-1-66)
Supplement: Additional file 1 — Search strategy and search terms. [file 2046-4053-1-66-S1.docx]

**Additional file 1. Search strategy and search terms**

The following sources were used to identify the relevant quantitative studies:

- Electronic databases

i) Cochrane Collaboration central register (CENTRAL/CCTR) (available through Cochrane library)

ii) Effective practice and organisation of care (EPOC) (Cochrane library)

iii) Database of Abstracts of Reviews for Effectiveness (DARE) (Cochrane library)

iv) Biomedical science databases

a) PubMed/MEDLINE (available through OVID)

b) Embase (available through NHS Evidence Health Information Resources/ NHS library)

c) CINAHL (Cumulative Index to Nursing and Allied Health Literature) (NHS Library)

d) PEDro (physiotherapy evidence database)

e) PsycINFO (psychological, social, behavioural and health sciences) (available through OVID)

f) AMED (Allied and Complementary Medicine) (NHS Library)

g) British Nursing Index (BNI) (NHS Library)

h) Research registers UK, USA

j) Applied Social Sciences Index and Abstracts (ASSIA) (available through ProQuest (part of Cambridge Information Group) - CSA Illumina web-based information system)

k) Education Resources Information Centre (ERIC) seminal

l) Social Science Citation Index (available through Web of Science) – forward citation tracking from identified “seminal” papers

The search terms were **(**Pulmonary Disease, Chronic Obstructive) OR (Lung Diseases, Obstructive) OR (chronic obstructive adj (pulmonary or lung or airway*) adj (disease* or obstruction or limitation*) OR (Emphysema) OR (Pulmonary Emphysema) OR (Bronchitis, Chronic) OR (Chronic Bronchitis) AND (Pulmonary rehabilitation) AND (Health Education/ or Patient Education as Topic/) OR (Health or patient) adj educat*) OR (Self Care) OR (self adj manage*) OR (psych* or behav*) adj (educat* or manage*) OR (expert adj patient) OR (self help) AND (Patient Participation) OR (patient* or particip*) adj (participat* or attend* or attitude* or motiv* or satisf* or involve* or accept* or refuse* or uptake or recruit* or rate*) OR (Consumer Participation) OR (Patient Acceptance of Health Care) OR (Patient Satisfaction/ or Attitude to Health/ or Aged/) OR Patient Dropouts) OR (drop out* or non attend* or barrier* or non participat*) OR (Program Evaluation) OR (programme evaluation) OR (Self Concept/ or Self Efficacy) OR (self-efficacy) OR (Cognition Disorders/ or Socioeconomic Factors/ or Depression) OR (Predict* adj attend*) OR (Health Services Accessibility) OR (complet* adj rate*).
