# Supplementary material for: Reporting participation rates in studies of non-pharmacological interventions for patients with chronic obstructive pulmonary disease: a systematic review
Source: Syst Rev. 2012 Dec 29;1:66. doi: 10.1186/2046-4053-1-66 (PMC3563605; doi:10.1186/2046-4053-1-66)
Supplement: Additional file 2 — Participant flow data and participation rates of three interventions. [file 2046-4053-1-66-S2.doc]

| **Additional file 2. Participant flow data and participation rates of three interventions** | | | | | | | | | | | | | | | | | | | | | | | | |
| --- | --- | --- | --- | --- | --- | --- | --- | --- | --- | --- | --- | --- | --- | --- | --- | --- | --- | --- | --- | --- | --- | --- | --- | --- |
| **Studies of pulmonary rehabilitation programmes, n= 31** | | | | | | | | | | | | | | | | | | | | | | | | |
|  | **Participant flow data** | | | | | | | | | | | | **Quality assessment** | | | **Participation rates** | | | | | | | | |
| **Author (year) study design** | **Potential numbers identified, n = 4** | | | **Numbers assessed, n = 12** | | | **Numbers eligible,**  **n = 21** | | | **Numbers included and randomised, n (I, C)** | | | **a, b, ci, cii, d** | | | **SPR,**  **n (%)** | | | **SDR,**  **n (%)** | | | **IDR,**  **n (%)** | | |
| Prince (1989)  RCT | not reported | | | not reported | | | not reported | | | 31 (13, 18) | | | 7, 1, 5, 3, no | | | n/c | | | 6 (19) | | | 2 (15) | | |
| Goldstein (1994)  RCT | not reported | | | 244 | | | 126 | | | 89 (45, 44) | | | 11, 1, 4, 3, no | | | 89 (71) | | | 11 (12) | | | 7 (16) | | |
| Reardon (1994)  RCT | not reported | | | not reported | | | not reported | | | 20 (10, 10) | | | 11, 1, 6, 3, no | | | n/c | | | 0 | | | 0 | | |
| Wijkstra (1994)  RCT | not reported | | | not reported | | | not reported | | | 45 (30, 15) | | | 10, 1, 5, 2, no | | | n/c | | | 2 (4) | | | 2 (7) | | |
| Ries (1995)  RCT | not reported | | | 352 | | | 128 | | | 119 (57, 62) | | | 9, 2, 6, 6, no | | | 119 (93) | | | 70 (59) | | | 31 (54) | | |
| Strijbos (1996)  RCT | not reported | | | not reported | | | 50 | | | 50 (18, 17, 15) | | | 10, 1, 4, 2, no | | | 50 (100) | | | 9 (18) | | | 5 (14) | | |
| Wedzicha (1998)  RCT | not reported | | | not reported | | | not reported | | | 126: moderate -66 (33, 33)  severe-60 (30, 30) | | | 10, 2, 5, 4, no | | | n/c | | | 17 (13) | | | 16 (25) | | |
| Ringbaek (2000)  RCT | not reported | | | not reported | | | 130 | | | 45 (24, 21) | | | 10, 1, 5, 5, no | | | 45 (35) | | | 7 (16) | | | 7 (29) | | |
| Guell (2000)  RCT | not reported | | | not reported | | | 65 | | | 60 (30, 30) | | | 10, 1, 5, 3, no | | | 60 (92) | | | 13 (22) | | | 6 (20) | | |
| Finnerty (2001)  RCT | not reported | | | 108 | | | 100 | | | 100 (50, 50) | | | 10, 1, 6, 2, no | | | 65 (65) | | | 16 (25) | | | 4 (11) | | |
| Green (2001)  RCT | not reported | | | not reported | | | not reported | | | 44 (23, 21) | | | 8, 1, 3, 1, no | | | n/c | | | 0 | | | 0 | | |
| White (2002)  RCT | 160 | | | 116 | | | 107 | | | 103 (54, 49) | | | 11, 2, 6, 4, yes | | | 103 (96) | | | 15 (15) | | | 9 (17) | | |
| de Godoy (2003)  RCT | not reported | | | not reported | | | 39 | | | 30 (14, 16) | | | 11, 1, 5, 5, no | | | 30 (77) | | | 0 | | | 0 | | |
| Oh (2003)  RCT | not reported | | | not reported | | | 34 | | | 34 (19, 15) | | | 10, 1, 5, 2, no | | | 34 (100) | | | 11 (32) | | | 4 (21) | | |
| Man (2004)  RCT | not reported | | | 69 | | | 52 | | | 42 (21, 21) | | | 11, 1, 5, 5, yes | | | 42 (81) | | | 8 (19) | | | 2 (10) | | |
| Lindsay (2005)  RCT | not reported | | | not reported | | | 50 | | | 50 (25, 25) | | | 10, 1, 5, 4, yes | | | 50 (100) | | | 9 (18) | | | 4 (16) | | |
| Boxall (2005)  RCT | not reported | | | not reported | | | not reported | | | 60 (30, 30) | | | 10, 1, 5, 3, yes | | | n/c | | | 14 (23) | | | 5 (18) | | |
| Na (2005)  Quasi-experimental | not reported | | | not reported | | | 43 | | | 43 (25, 18) | | | 10, 1, 6, 1, yes | | | 43 (100) | | | 20 (47) | | | 5 (20) | | |
| Sewell (2006)  RCT | not reported | | | not reported | | | not reported | | | 100 (50, 50) | | | 10, 1, 6, 4, yes | | | n/c | | | 29 (29) | | | 9 (18) | | |
| Guell (2006)  RCT | not reported | | | not reported | | | not reported | | | 40 (20, 20) | | | 9, 1, 5, 3, no | | | n/c | | | 5 (13) | | | 2 (10) | | |
| Resqueti (2007)  RCT | not reported | | | not reported | | | 42 | | | 38 (19, 19) | | | 10, 1, 5, 4, no | | | 38 (90) | | | 9 (24) | | | 5 (26) | | |
| Karapolat (2007)  RCT | not reported | | | not reported | | | not reported | | | 49 (27, 22) | | | 9, 1, 5, 2, no | | | n/c | | | 4 (8) | | | 1 (4) | | |
| Sridhar (2008)  RCT | 1247 | | | 574 | | | 297 | | | 122 (61, 61) | | | 9, 2, 4, 5, yes | | | 122 (42) | | | 18 (15) | | | 6 (10) | | |
| Barakat (2008)  RCT | not reported | | | 80 | | | 80 | | | 80 (40, 40) | | | 9, 2, 6, 4, no | | | 80 (100) | | | 9 (11) | | | 5 (13) | | |
| Carr (2009)  RCT | not reported | | | 364 | | | 94 | | | 60 included, 34 (17, 17) | | | 10, 2, 5, 4, yes | | | 34 (36) | | | 5 (15) | | | 1 (6) | | |
| Theander (2009)  RCT | 64 | | | 55 | | | 30 | | | 30 (15, 15) | | | 10, 2, 4, 5, yes | | | 30 (100) | | | 4 (13) | | | 3 (20) | | |
| van Wetering (2009)  RCT | not reported | | | 355 | | | 199 | | | 199 (102, 97) | | | 10, 2, 4, 4, yes | | | 199 (100) | | | 41 (21) | | | 18 (19) | | |
| Ghanem (2010)  RCT | not reported | | | not reported | | | 50 | | | 45 (30, 15) | | | 10, 1, 5, 5, yes | | | 39 (78) | | | 0 | | | 0 | | |
| Liddell (2010)  Pilot RCT | 36 on waiting list | | | 36 | | | 30 | | | 30 (15, 15) | | | 9, 1, 5, 4, no | | | 30 (100) | | | 10 (33) | | | 4 (27) | | |
| Seymour (2010)  RCT | not reported | | | not reported | | | not reported | | | 60 (30, 30) | | | 10, 1, 5, 4, yes | | | n/c | | | 7 (12) | | | 7 (23) | | |
| Zakrisson (2011)  Quasi-experimental | not reported | | | 1828 | | | 176 | | | 176 (83, 93) | | | 10, 1, 3, 1, yes | | | 176 (100) | | | 12 (7) | | | 0 | | |
| **Studies of self-management programmes, n=21** | | | | | | | | | | | | | | | | | | | | | | | | |
| **Author (year) study design** | | | **Potential numbers identified,**  **n = 4** | | | **Numbers assessed,**  **n = 10** | | | **Numbers eligible,**  **n = 19** | | | **Numbers included and randomised, n (I, C)** | | | **a, b, ci, cii, d** | | | **SPR,**  **n (%)** | | | **SDR,**  **n (%)** | | | **IDR,**  **n (%)** |
| Howland (1986)  Quasi-experimental | | | not reported | | | 1834 | | | 923 | | | 659 (254, 405) | | | 10, 0, 5, 2, no | | | 659 (71) | | | 121 (18) | | | 41 (16) |
| Cockcroft (1987)  RCT | | | not reported | | | not reported | | | 92 | | | 75 (42, 33) | | | 10, 3, 4, 3, no | | | 73 (79) | | | 11 (15) | | | 3 (8) |
| Sassi-Dambron (1995)  RCT | | | not reported | | | 497 | | | 98 | | | 98 (47, 51) | | | 10, 1, 5, 3, yes | | | 89 (91) | | | 13 (15) | | | 5 (11) |
| Zimmerman (1996)  Before-after | | | not reported | | | not reported | | | not reported | | | 10 | | | 8, 1, 3, 1, no | | | n/c | | | 0 | | | Same as SDR |
| Emery (1998)  RCT | | | not reported | | | not reported | | | 92 | | | 79 (29, 25, 25) | | | 10, 1, 7, 5, no | | | 79 (86) | | | 6 (8) | | | 4 (14) |
| Bourbeau (2003)  RCT | | | not reported | | | not reported | | | 469 | | | 191 (96, 95) | | | 10, 1, 6, 5, yes | | | 191 (41) | | | 26 (14) | | | 10 (10) |
| Monninkhof (2003)  RCT | | | 615 | | | not reported | | | 509 | | | 248 (127, 121) | | | 11, 1, 5, 5, yes | | | 248 (41) | | | 12 (5) | | | 5 (4) |
| Rea (2004)  RCT | | | not reported | | | 700 | | | 158 | | | 135 (83, 52) | | | 10, 1, 4, 5, yes | | | 135 (85) | | | 18 (13) | | | 12 (14) |
| Kara (2004)  RCT | | | not reported | | | not reported | | | 60 | | | 60 (30, 30) | | | 11, 1, 4, 4, no | | | 60 (100) | | | 0 | | | 0 |
| Coultas (2005)  RCT | | | 2120 | | | 535 | | | 217 | | | 217 (72, 72, 73) | | | 10, 2, 5, 5, yes | | | 217 (100) | | | 66 (30) | | | 44 (31) |
| Casas (2006)  RCT | | | not reported | | | 850 | | | 160 | | | 155 (65, 90) | | | 10, 3, 7, 4, yes | | | 155 (97) | | | 35 (23) | | | 17 (26) |
| Nyugen (2008)  Pilot RCT | | | not reported | | | 173 | | | 84 | | | 50 (26, 24) | | | 10, 2, 5, 5, no | | | 50 (60) | | | 11 (22) | | | 7 (27) |
| Kheirabadi (2008)  RCT | | | not reported | | | not reported | | | 59 | | | 42 (21, 21) | | | 10, 1, 4, 4, no | | | 42 (71) | | | 0 | | | 0 |
| Efraimmson (2008)  RCT | | | 110 | | | 110 | | | 62 | | | 62 (26, 26) | | | 10, 1, 4, 4, no | | | 52 (84) | | | 0 | | | 0 |
| Effing (2009)  RCT | | | not reported | | | not reported | | | 421 | | | 153 (77, 76) | | | 10, 1, 5, 5, yes | | | 153 (36) | | | 11 (7) | | | 11 (16) |
| Khdour (2009)  RCT | | | not reported | | | not reported | | | 295 | | | 173(86, 87) | | | 10, 1, 4, 4, yes | | | 173 (59) | | | 30 (17) | | | 15 (17) |
| Moore (2009)  Pilot RCT | | | not reported | | | 40 | | | 33 | | | 27 (14, 13) | | | 10, 1, 5, 3, no | | | 27 (82) | | | 7 (26) | | | 4 (29) |
| Koff (2009)  Pilot RCT | | | not reported | | | not reported | | | not reported | | | 40 (20, 20) | | | 11, 0, 4, 4, yes | | | n/c | | | 2 (5) | | | 1 (5) |
| Hill (2010)  RCT | | | 131 | | | 110 | | | 100 | | | 100 (55, 45) | | | 10, 3, 5, 4, yes | | | 100 (100) | | | 7 (7) | | | 5 (9) |
| Ninot (2011)  RCT | | | not reported | | | 101 | | | 61 | | | 45 (23, 22) | | | 11, 1, 4, 4, yes | | | 45 (74) | | | 7 (16) | | | 1 (4) |
| Taylor (2012)  Pilot RCT | | | not reported | | | not reported | | | 507 | | | 116 (78, 38) | | | 9, 2, 5, 6, no | | | 116 (23) | | | 25 (22) | | | 47 (60) |
| **Studies of health education programmes, n=4** | | | | | | | | | | | | | | | | | | | | | | | | |
| **Author (year) study design** | | **Potential numbers identified,**  **n = 1** | | | **Numbers assessed,**  **n = 0** | | | **Numbers eligible,**  **n = 3** | | | **Numbers included and randomised, n (I, C)** | | | **a, b, ci, cii, d** | | | **SPR, n (%)** | | | **SDR, n (%)** | | | **IDR, n (%)** | |
| Littlejohn  (1991)  RCT | | not reported | | | not reported | | | 166 | | | 152 (73, 79) | | | 11, 1, 6, 4, yes | | | 152 (92) | | | 19 (13) | | | 5 (7) | |
| Petty (2006)  RCT | | more than 500 | | | not reported | | | not reported | | | 214 (72, 69, 73) | | | 10, 3, 3, 5, no | | | n/c | | | 40 (19) | | | 21 (29) | |
| Rice (2010)  RCT | | not reported | | | not reported | | | 1739 | | | 743 (372, 371) | | | 10, 1, 5, 4, yes | | | 743 (43) | | | 84 (11) | | | 36 (10) | |
| Lemmens (2010)  Before-after | | not reported | | | not reported | | | 259 | | | 259 | | | 10, 2, 3, 1 no | | | 189 (73) | | | 39 (21) | | | Same as SDR | |
| **Key:**  RCT – Randomised Controlled Trial  SPR – Study participation rate  SDR – Study dropout rate  IDR – Intervention dropout rate  n – numbers  I – Intervention  C – Control  n/c – not calculable  a – Reporting (ten questions), maximum score out of 11  b – External validity (three questions), maximum score about of 3  ci – Internal validity-bias (seven questions), maximum score out of 7  cii – Internal validity-confounding (selection bias) (six questions), maximum score out of 6  d – Power (one question), response: yes/no | | | | | | | | | | | | | | | | | | | | | | | | |
